# Telomere Length, Telomerase Activity, and Vaginal Microbiome in Patients with HPV-Related Precancerous Lesions

**DOI:** 10.3390/ijms25158158

**Published:** 2024-07-26

**Authors:** Ewa Boniewska-Bernacka, Anna Pańczyszyn, Grzegorz Głąb, Anna Goc

**Affiliations:** 1 Department of Biology and Genetics, Institute of Medical Sciences, Faculty of Medicine, University of Opole, Oleska 48, 45-052 Opole, Poland; apanczyszyn@uni.opole.pl (A.P.); anna.goc@uni.opole.pl (A.G.); 2 Department of Pathomorphology, Institute of Medical Sciences, Faculty of Medicine, University of Opole, Oleska 48, 45-052 Opole, Poland; grzegorz.glab@uni.opole.pl

**Keywords:** telomeres, telomerase, biocenosis, human papillomavirus, cervical smear

## Abstract

Persistent high-risk human papillomaviruses (HR HPVs) infection leads to the development of squamous intraepithelial lesions in cervical cells that may lead to cancer. The telomere length, telomerase activity, and species composition of the vaginal microbiome may influence the dynamic of changes and the process of carcinogenesis. In the present study, we analyze relative telomere length (RTL), relative *hTERT* expression (gene for the telomerase component—reverse transcriptase) in cervical smear cells and vaginal microbiomes. Total RNA and DNA were isolated from tissue samples of 109 patients from the following groups: control, carrier, low-grade or high-grade squamous intraepithelial lesion (L SIL and H SIL, respectively), and cancer. The quantitative PCR method was used to measure telomere length and telomerase expression. Vaginal microbiome bacteria were divided into community state types using morphotype criteria. Significant differences between histopathology groups were confirmed for both relative telomere length and relative *hTERT* expression (*p* < 0.001 and *p* = 0.001, respectively). A significant difference in RTL was identified between carriers and H SIL (*p* adj < 0.001) groups, as well as between carriers and L SIL groups (*p* adj = 0.048). In both cases, RTL was lower among carriers. The highest relative *hTERT* expression level was recorded in the H SIL group, and the highest relative *hTERT* expression level was recorded between carriers and the H SIL group (*p* adj < 0.001). A correlation between genotype and biocenosis was identified for genotype 16+A (*p* < 0.001). The results suggest that identification of HPV infection, telomere length assessment, and *hTERT* expression measurement together may be more predictive than each of these analyses performed separately.

## 1. Introduction

Telomeres are nucleoprotein structures located at the ends of the chromosomes of eukaryotic cells. They protect chromosomes against degradation and fusion and participate in the spatial organization of the cell nucleus, as well as regulate the transcription of genes located in subtelomeric areas. In humans, they consist of tandem repeats of the 5′-TTAGGG-3′ sequence combined with a complex of specialized proteins [1]. In somatic cells, telomeres shorten with each replication cycle. This phenomenon is the result of DNA replication and processing at the ends of chromosomes, which is necessary to create a functional telomere. Critical shortening of telomeres results in cell senescence or the initiation of apoptosis [2]. Telomeres may be lengthened by the telomerase [3], an enzyme composed of a catalytic subunit—reverse transcriptase (hTERT), non-coding RNA (TERC), which serves as a template for telomere elongation, and proteins that stabilize the enzyme binding to DNA. In differentiated somatic cells, its activity remains at a very low level or is undetectable. In turn, in cancer cells, telomerase is usually expressed at a high level, which allows for unlimited proliferation [4]. The level of *hTERT* expression is an indicator of carcinogenesis in some cancers and may indicate cancer metastases after tumor resection [5].

Telomerase activation is an important step in cell immortalization during infection with HR HPVs, leading to the development of cervical cancer [6,7]. HPV is transmitted by direct contact, mainly through sexual behavior. The most oncogenic types, 16 and 18, are responsible for a 200-fold higher risk of invasive cervical cancer compared to other HPV types [8]. HR HPV infection can cause intraepithelial lesions (SILs), which are divided into two stages: low-grade or high-grade SILs (L SILs and H SILs, respectively) [8,9]. Women with HR HPVs and H SILs have a higher risk of developing cervical cancer compared to women without HR HPVs [10]. The dynamics of the development of malignant tumors may vary among different women and may persist for several years [11]. Commonly employed screening techniques, such as cytological examination, colposcopy, and molecular diagnostic techniques, facilitate the preclinical detection of pathological alterations in the cervix during the pre-invasive stage, when local excision provides fully efficacious treatment [12]. The increased risk of cancer for individuals with HR HPV infection was confirmed by molecular biology methods [13]. Nevertheless, molecular biomarkers useful for assessing changes at the preclinical stage that would identify women with high risk of rapid disease progression are still being sought [14].

HR HPVs were divided into three groups according to the genotype and the associated risk level [13,15,16]. The most common, HPV type 16 (group 16), is responsible for 50% of H SILs, while 45% of cases are caused by types 18/31/33/52/58/45 (group A). In turn, 5% of H SILs are caused by types 51/39/68/56/59/66/35 (group B).

The presence of HPV infection is correlated with the risk of cervical cancer but does not mean that the cancer appears. The main natural protection against HR HPVs is the physiological vaginal microbiome [17,18]. The *Lactobacillus* spp. present in vaginal mucus are not a homogenous group; they are divided into five community-state types (CSTs) [19,20]. In particular, *Lactobacillus crispatus* (CSTs I), *Lactobacillus gasseri* (CSTs II) and *Lactobacillus jenseni* (CSTs V) are considered to be optimal for microbiomes because of lactobiocins and biosurfactant secretion, which may interrupt viral infiltration into squamous cells [19,21]. Although *Lactobacillus iners* (CSTs III) is a physiological component of the vaginal microflora, it is not considered to be optimal due to its lack of ability to produce lactobiocins and hydrogen peroxide [22]. The absence of *Lactobacillus* spp. promotes the development of other components of the vaginal microbiome such as *Gardnerella vaginalis, Prevotella, Atobium, Megasphera, Peptostreptococcus,* and *Sneathia*, known as CSTs IV [23]. Direct microscopy of the cervico-vaginal smears allows for classification of the CSTs using morphotype criteria: optimal forms (*L crispatus, L. gasseri, L. jenseni*), suboptimal (*L. iners* CSTs III) and dysbiosis (CSTs IV) [19,23]. The complex diagnosis including HPV genotype identification, the actual status of the vaginal microbiome as well as the expression of *hTERT*, and telomere length may provide information about the risk of carcinogenesis and may be helpful in cervical cancer identification at a very early stage [24]. The aim of our study was to analyze the relative telomere length, the relative expression of *hTERT* in cervical smear cells, and the vaginal microbiome.

## 2. Results

### 2.1. Relative Telomere Length (RTL) and Relative hTERT Expression in Different Histopathology Groups

**Figure 1:**
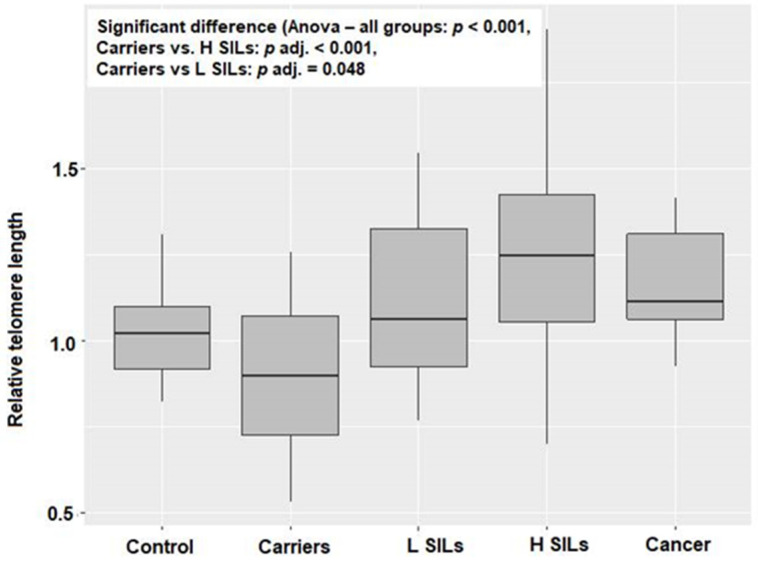
RTL of analyzed histopathology groups.

**Figure 2:**
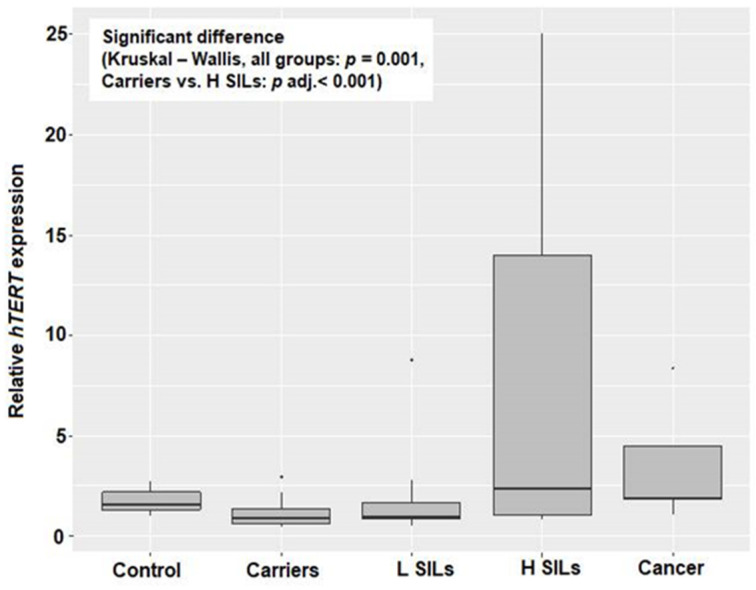
Relative *hTERT* depending on histopathology group.

RTL and relative *hTERT* expression were measured for groups of patients. Significant differences between histopathology groups were confirmed for both relative telomere length (RTL) and relative *hTERT* expression (*p* < 0.001 and *p* = 0.001, respectively; Table 1, Figure 1 and Figure 2). Based on pairwise comparisons, a significant difference in RTL was identified between carriers and H SILs (*p* adj < 0.001), as well as between carriers and L SILs (*p* adj = 0.048), and in both cases RTL was lower among carriers. A statistically significant difference in relative *hTERT* expression was also observed between carriers and the H SILs group (*p* adj < 0.001).

The obtained results indicate significantly higher relative *hTERT* expression in the H SILs group and, consequently, longer telomeres in cervical epithelial cells in patients of this group.

### 2.2. The Biocenosis of Different Histopathology Groups

Histopathology outcomes were significantly dependent on biocenosis type, *p* < 0.001 (Table 2). The proportion of patients with an optimal outcome of biocenosis was the highest among the control group (83.3%) and lowered with the worsening outcome of histopathology to 15.2% (n = 7) among patients with H SILs. There were no patients with optimal biocenosis in the cancer group. The distribution of *L. iners* was the highest among patients with L SILs and H SILs (64.3%, n = 9 and 58.7%, respectively). There were no patients with dysbiosis in the control group. In patients of the L SILs and carrier groups, dysbiosis was rarely detected (16.7%, n = 5 and 14.3%, n = 2, respectively). Dysbiosis was more frequent among patients with H SILs (26.1%), but the highest percentage of dysbiosis was found among patients with cancers (71.4%, n = 5).

The relationship between the histopathological group of patients, the biocenosis, and HPV genotype is described in Table 3. The majority of patients in the control group had optimal biocenosis. In other groups, the percentage of these patients was lower. Among the cancer group, no patient had optimal biocenosis; all of them had *L. iners* and dysbiosis. In the control group, there were no patients with HPV infection of the 16+A genotype. The prevalence of such patients increased in other groups. In the cancer group, 100% of patients were infected with the HPV 16+A genotype. In turn, in the control group, there were no patients with HPV genotype B infection, which concerned mostly the carrier group. The percentage of patients with this infection decreased in subsequent groups.

The relationship between genotype, biocenosis, and RTL/relative *hTERT* expression was also investigated (Table 4). A significant correlation between the HPV 16+A genotype and biocenosis was identified (*p* < 0.001). The percentage of patients with optimal biocenosis was lower in the group with HPV genotype 16+A compared to the group without this HPV genotype (17.9% vs. 72.0%). The proportion of *L. iners* in patients with the HPV genotype 16+A was higher than in patients without genotype 16+A (53.6% vs. 28.0%, n = 7). Dysbiosis was more common in patients with the HPV 16+A genotype than in patients without this HPV genotype (28.6% vs. 0.0%). The incidence of HPV genotype B was not related to the type of biocenosis (*p* = 0.055).

RTL varied according to the presence of HPV genotype 16+A with the higher outcome of RTL among patients with the 16+A genotype compared to patients with no HPV 16+A genotype, MD = 0.13 CI95 [0.03; 0.22], *p* = 0.008. RTL was not associated with the incidence of HPV genotype B. The relative expression of *hTERT* was lower in patients with HPV genotype B compared to patients without this genotype, MD = −0.58 CI95 [−1.23; −0.03], *p* = 0.033. Telomerase gene activity was not differentiated with the incidence of genotype 16+A (*p* = 0.637).

There was no significant correlation between age and relative telomere length (RTL) (*p* > 0.05), or between age and relative *hTERT* expression (*p* > 0.05).

## 3. Discussion

Although high-risk human papillomavirus infection is considered a leading cause of cervical cancer, not all infected women develop this cancer. There are a growing number of reports which indicate that factors like telomere length, telomerase activity, and biocenosis may have an impact on the carcinogenesis process in the case of HR-HPV infection.

The presented study included specimens from 109 women. They were divided into five groups depending on their histopathology analysis results. The analysis of every specimen included HPV genotype identification and vaginal biocenosis assessment, as well as telomere length and telomerase expression in epithelial cells of the cervix. In our research, the longest telomeres were in the H SILs group, where there was a significant relationship between *hTERT* expression and telomere length. As a result of high telomerase activity, we observed telomeres lengthening in epithelial cells of the cervix. This result is consistent with the previous one observed by Moreno-Acosta et al. [25] who indicated that *hTERT* expression increases significantly with the progression of premalignant cervical lesions and presents as an early event in the course of cervical cancer. We observed the tendency of telomeres shortening in the cancer group. This may confirm previous research which demonstrated that telomeres lengthen in advanced cancers, and that their length stabilizes after the stage of shortening and intensive cell proliferation that is usually observed in the advanced carcinogenesis process [26,27].

The *hTERT* expression was correlated with the higher risk of precancerous lesions in women infected with HPV genotype 16+A. Also, others [28,29] observed a higher level of telomerase activity in both precancerous lesions and cancer, with concomitant undetectable or extremely low levels of *hTERT* expression in normal tissue.

Several studies demonstrated a high frequency of HR-HPV infection in women with H SILs and a low frequency of HPV infection in women without any lesions [8,30]. Similarly, in our study, 94.5% of women with H SILs and 100% of women with cancer were infected with HPV genotype 16+A. There was no incidence of HPV infection in the control group.

The results suggest that there is a higher probability that women with HPV genotype 16+A, higher *hTERT* expression, and longer telomeres will develop H SILs. Previously, Chen et al. [31] proved that women with HPV 16 infections with shorter telomeres have a higher risk of cervical cancer and Molano [28] showed a correlation between telomerase activity and malignant cervical lesions.

The published data indicate that there is a relationship between the vagina microbiome, HPV infection [32], and squamous intraepithelial lesions [18,33,34]. The optimal microbiome with dominance of *Lactobacillus* spp. including *L. gasseri*, *L. jensenii,* and *L. crispatus*, seems to protect against HPV infection [34,35,36]. In turn, Oh et al. [37] indicated that the cervix microbiome with an advantage of *Atopobium vaginae*, *Gardnerella vaginalis*, and *L. iners* resulted in a six times higher risk of SILs. Similarly, Mortaki [32] suggested that microorganisms like *Sneathia sp.*, *Anaerococcus tetradius, Peptostreptococcus, Fusobacterium i Gardnerella vaginalis*, together with a low number of *Lactobacillus* strains, are correlated with higher risk of HPV infection and lower chance of remission. Our research provided similar results. SILs were significantly related to biocenosis (*p* < 0.001). The number of patients with optimal microbiome was the highest in the control group and decreased with the deterioration of histopathology results. There were no patients with optimal biocenosis in the cancer group. There was a correlation between the HPV 16+A genotype and biocenosis.

In the control group, no dysbiosis was observed. Dysbiosis was rare in the carrier and L SIL groups, but its frequency increased with the worsening of histopathological results. Among patients with HPV genotype 16+A infections, the frequency of dysbiosis was higher compared to patients not infected with these HP genotypes.

Our study demonstrated that *L. iners* was a common component of microbiomes in patients with dysbiosis. The highest number of patients with *L. iners* was in L SIL and H SIL groups. Also, *L. iners* was more frequent in patients with an HPV genotype 16+A infection than in patients without HPV genotype 16+A. Although *L. iners* is considered a physiological component of the microbiome, it is not an optimal one [17,21]. A few reports suggest that *L. iners* may be associated with cervical dysplasia [37,38], and its presence is connected with microbiome disorders caused by HPV [39,40].

The literature reports that the cervical microbiome has the potential to serve as a biomarker to assess the risk of cervical cancer progression [21,33,36]. In our study, we performed a sensitivity and specificity analysis regarding the prognostic quality of a positive biocenosis result (dysbiosis/*L.iners*) for diagnosing subsequent grades of histopathological results (Supplementary Table S1). In no case did we achieve high prognostic quality. The sensitivity increased with more advanced histopathology grades, but the specificity decreased. The accuracy analysis indicated that it was not difficult to distinguish the H SIL vs. L SIL groups based on the microbiomes (70%). It seems that patients with H SILs have an unfavorable prognosis. There is a high risk that their precancerous lesions could transform directly into invasive cancer. Hence, such patients should be carefully monitored. Detailed diagnostics should also be introduced in cases of patients with L SILs with co-occurrence of dysbiosis or *L. iners.*

In conclusion, our results suggest that identification of HPV infection, telomere length assessment, and *hTERT* expression measurement together may have a higher predictive significance than each of these analyses separately. However, our research has some limitations, especially with regard to the small number of patients with cancer and a lack of correlation between patient age and RTL. This may be due to the fact that RTL was measured in epithelial cells of the cervix, which were impacted by many factors, including not only age, but also HPV infection and microbiome.

In summary, this is the second epidemiological study that indicates an independent association of telomerase activity with HPV as a risk factor for H SILs. It is also the first study which demonstrates the association of telomerase activity, RTL, and HPV with vaginal biocenosis.

## 4. Materials and Methods

### 4.1. Patient Samples

The study included 109 cervical swab samples from patients of the Gynecological and Obstetric Diagnostic Center in Opole (Poland). In all cases, after colposcopic target biopsy followed by histopathological examinations in a certified Diagnostica Consilio Laboratory in Łódź (Poland), the patients were divided into the following groups: control, carriers, L SILs, H SILs, and cervical cancers. Molecular detection of HPV genotypes was performed using a validated test (Abbott RealTime High Risk HPV Amplification Kit, Des Plaines, IL, USA) and nested PCR. Because HR HPVs group 16 and group A are responsible for 95% of H SILs, they are combined in our study into one group (16+A). The second group consists of HR HPVs of genotype B. The clinical characteristics of the patients are presented in Table 5.

### 4.2. Ethics Approval and Consent to Participate

This study was approved by the local Bioethics Committee. The patients were informed of the purpose of the study and the use of their materials for scientific research. All patients gave their written informed consent and completed the information survey. All procedures performed in this study were in accordance with the ethical standards of the institutional and national regulations and with the Helsinki Declaration.

### 4.3. Biocenosis Examination

Vaginal microbiome components were identified in direct cervico-vaginal smears with a cervix brush using Phase-contrast microscope Zeiss AxioStar in 500× magnification and captured by digital HD camera in computer records, and with colposcopic images of the uterine cervix (Carl Zeiss, Oberkochen, Germany). We use a digital colposcope Medicom V1000 HDMI (Medicom, Zabrze, Poland). Vaginal microbiome bacteria were divided into Community State-types using the morphotype criteria published by Gary Ventolini [19,23,41,42]. An optimal microbiome was defined when *L. crispatus, L. gasseri*, *and L. jenseni* were present in the vaginal direct smear. The *Lactobacillus iners* (CSTsIII) and dysbiosis (CSTsIV) were treated as other groups (suboptimal and pathology).

### 4.4. Nucleic Acid Extraction and cDNA Synthesis

DNA was isolated using a GeneMATRIX Swab-Extract DNA Purification Kit (Eurx, Gdańsk, Poland) according to the manufacturer’s instructions. Extracted DNA was then quantified using a BioSpectrometer (Eppendorf, Hamburg, Germany). Total RNA from individual patients was extracted using the TRIzol^TM^ Reagent (Thermo Fisher Scientific, Waltham, MA, USA) following the manufacturer’s instructions. RNA was reverse transcribed using the iScript cDNA Synthesis Kit (Bio-Rad Laboratories, Hercules, CA, USA) in a final volume of 20 µL as follows: 5 min at 25 °C and 20 min at 46 °C. The reverse transcriptase was inactivated by heating at 95 °C for 1 min.

### 4.5. Telomere Length Measurement by Monochrome Multiplex Quantitative PCR Method (MMQPCR)

Telomere length was determined using the multiplex quantitative PCR (MMQPCR) according to the procedure described by Cawthon [43], with minor modifications. The 3-fold dilution series (60 ng to 0.74 ng) of genome DNA was used as a reference sample to prepare the standard curves. All experimental and standard samples were run in triplicate. Each reaction well contained 2 μL of DNA (10 ng), 2× SYBR Green PCR Master Mix (Bio Rad, Hercules, CA, USA), two pairs of primers (telg and telc primer [43]—500 nM; albugcr1 and albdgcr2 primer [44]—900 nM; Table 6) and water to a final volume of 10 μL. The thermal cycling profile was as follows: 15 min at 95 °C, 2 cycles of 15 s at 94 °C, 15 s at 49 °C, 35 cycles of 15 s at 94 °C, 10 s at 62 °C, 15 s at 74 °C with signal acquisition (signal for telomeres), 10 s at 84 °C, and 15 s at 88 °C with signal acquisition (signal for albumin). After thermal cycling and raw data collection, CFX Manager Software (Bio Rad CFX Maestro 1.1 version 4.1.2433.1219) was used to generate two standard curves for each plate, one for telomeres and the second one for the reference gene—albumin. The efficiency of the reaction was equal for telomeres and albumin, and it was no lower than 90%. After the run was complete, the MyiQ software (Bio Rad CFX Maestro 1.1 version 4.1.2433.1219) was used to determine the T (telomere) and S (single-copy gene) values. Therefore, a ratio between products of telomeres and albumin (T/S) represents a quantity that is proportional to the average telomere length per cell and represents relative telomere length (RTL). The average telomere length of the sample with a T/S of >1.0 is higher than that of the standard DNA; the average telomere length of the sample with a T/S of 1.0 is lower than that of the standard DNA.

### 4.6. Quantitative RT-PCR of hTERT

The expression of *hTERT* (Hs05045220_g1) and *GAPDH* (Hs03929097_g1) genes was measured by qRT-PCR, based on the TaqMan methodology (Thermo Fisher Scientific, USA) using a Bio-Rad CFX96 real-time PCR system. PCR reactions were processed to a final volume of 10 µL containing 5 µL of 2× TaqMan Universal PCR Master Mix (Thermo Fisher Scientific, Waltham, MA, USA); 0.5 µL TaqMan assay (20×), 2 µL of sample cDNA (200 ng), and 2.5 µL of RNAse-free water. The thermal cycling profile was as follows: 2 min at 50 °C, 10 min at 95 °C, and 40 cycles of the following: 15 s at 95 °C and 1 min at 60 °C. The standard curve method has been applied to analyze *hTERT* expression. The 10-fold dilution (990 pg to 99 pg) of genome DNA was used on every plate to prepare the standard curve separately for *hTERT* and *GAPDH*. For all experimental samples, the target quantity (*hTERT*) was determined from the standard curve and divided by the target quantity of the calibrator (*GAPDH*).

### 4.7. Statistical Analysis

Statistical analysis was performed with R software, version R-4.1.2. Nominal variables were described with n (%); numeric variables were described with the mean ± SD or median (interquartile range), depending on distribution. Normality of distribution was verified with a Shapiro–Wilk test, skewness, and kurtosis. Variance homogeneity was checked with Levene’s test. For comparison of groups, the t-Student test, *t*-Welch test, Mann–Whitney U test, Anova, Kruskal–Wallis test, Pearson’s Chi-square test, or Fisher’s exact test were used, depending on variable types and satisfaction of assumptions. Pairwise comparisons were performed with Tukey’s test or Dunn’s test with Bonferroni adjustment, as appropriate. All statistical tests assumed α = 0.05. For assessment of prognostic quality sensitivity, specificity, PPV (positive prognostic value), NPV (negative prognostic value) and accuracy were calculated, along with 95% CI (based on https://www.medcalc.org/calc/diagnostic_test.php, 1 December 2023).

## Figures and Tables

**Table 1:** RTL and *hTERT* expression in different histopathology groups.

Variable	Control	Carriers	L SIL	H SIL	Cancers	*p*
Relative telomere length RTL, mean ± SD	1.03 ± 0.15	0.90 ± 0.21 ^ab^	1.12 ± 0.25 ^b^	1.25 ± 0.28 ^a^	1.17 ± 0.19	<0.001
Relative *hTERT* expression, median (IQR)	1.59 (1.34; 2.23)	0.87 (0.67; 1.41) ^c^	0.99 (0.88; 1.70)	2.39 (1.08; 14.02) ^c^	1.91 (1.89; 4.48)	0.001 ^1^

SD—standard deviation, IQR—interquartile range. Groups were compared with Anova analysis or the Kruskal–Wallis test ^1^, as appropriate. Pairwise comparisons were performed with Tukey’s test (for Anova outcome) or Dunn’s test with Bonferroni adjustment (for Kruskal–Wallis outcome). ^a–c^—pairs of groups with significant difference, based on pairwise comparisons (^a^: *p* adj < 0.001, ^b^: *p* adj = 0.048, ^c^: *p* adj < 0.001).

**Table 2:** The relation between biocenosis and histopathology group.

Biocenosis	Control (n = 12)	Carriers (n = 30)	L SILs (n = 14)	H SILs (n = 46)	Cancers (n = 7)	*p*
Optimal	10 (83.3)	13 (43.3)	3 (21.4)	7 (15.2)	0 (0.0)	<0.001
*L. iners*	2 (16.7)	12 (40.0)	9 (64.3)	27 (58.7)	2 (28.6)	
Dysbiosis	0 (0.0)	5 (16.7)	2 (14.3)	12 (26.1)	5 (71.4)	

Data are given as n (%). Comparison performed with Fisher exact test.

**Table 3:** Relationship between the histopathological group, biocenosis, and HPV genotype.

Biocenosis and HPV Genotype		Histopathology Groups [%]				
		Control	Carriers	L SILs	H SILs	Cancers
Biocenosis	Optimal	83.3	43.3	21.4	15.2	0
	*L. iners* and dysbiosis	16.7	56.7	78.6	84.8	100
HPV genotype	Genotype 16+A	0	69	92.9	94.5	100
	Genotype B	0	44.8	28.6	17.4	14.3

**Table 4:** Relationship between genotype, biocenosis and telomere length/relative *hTERT* expression.

Variable		Incidence of HPV Genotype		MD (95% CI)	*p*
		Yes	No		
**HPV genotype 16+A vs:**					
Biocenosis	Optimal	15 (17.9)	18 (72.0)	-	**<0.001 ^3^**
	*L. iners*	45 (53.6)	7 (28.0)		
	Dysbiosis	24 (28.6)	0 (0.0)		
Relative telomere length (RTL), mean ± SD		1.14 ± 0.30	1.01 ± 0.17	0.13 (0.03; 0.22)	**0.008 ^2^**
Relative *hTERT* expression, median (IQR)		1.49 (0.89; 4.47)	1.32 (1.03; 2.21)	0.17 (−0.36; 1.02)	0.637
**HPV genotype B vs:**					
Biocenosis	Optimal	12 (42.9)	21 (25.9)	-	0.055 ^3^
	*L. iners*	14 (50.0)	38 (46.9)		
	Dysbiosis	2 (7.1)	22 (27.2)		
Relative telomere length (RTL), mean ± SD		1.05 ± 0.31	1.13 ± 0.27	−0.08 (−0.20; 0.05)	0.227 ^1^
Relative *hTERT* expression, median (IQR)		1.03 (0.67; 1.70)	1.61 (0.99; 4.42)	−0.58 (−1.23; −0.03)	**0.033**

SD—standard deviation, IQR—interquartile range, MD—mean or median difference (yes vs. no), CI—confidence interval. Comparison performed with t-Student test ^1^, *t*-Welch test ^2^, Mann–Whitney U test and Pearson’s Chi-square test ^3^. Statistically significant differences are highlighted in bold.

**Table 5:** Characteristics of the study groups.

Variable	n	Statistics
Age, years, mean ± SD	109	37.31 ± 10.36
Histopathology group	109	
Control	12	11.0
Carriers	30	27.5
L SILs	14	12.8
H SILs	46	42.2
Cancers	7	6.4
Biocenosis	109	
Optimal	33	30.3
*L. iners*	52	47.7
Dysbiosis	24	22.0
HPV genotype	109	
Genotype 16+A	84	77.1
Genotype B	28	25.7
Relative telomere length (RTL), mean ± SD	107	1.11 ± 0.28
Relative *hTERT* expression, median (IQR)	66	1.39 (0.93; 2.68)

SD—standard deviation, IQR—interquartile range. Statistics presented as % unless indicated otherwise.

**Table 6:** Sequences of primers.

Primer	Sequence 5′–3′
telg	ACACTAAGGTTTGGGTTTGGGTTTGGGTTTGGGTTAGTGT
telc	TGTTAGGTATCCCTATCCCTATCCCTATCCCTATCCCTAACA
albugcr1	CGGCGGCGGGCGGCGCGGGCTGGGCGGCCATGCTTTTCAGCTCTGCAAGTC
albdgcr2	GCCCGGCCCGCCGCGCCCGTCCCGCCGAGCATTAAGCTCTTTGGCAACGTAGGTTTC

## Data Availability

The datasets analyzed during the current study are not publicly available because individual privacy could be compromised, but they are available from the corresponding author (Ewa Boniewska-Bernacka) on reasonable request.

## Supplementary Materials

The following supporting information can be downloaded at: https://www.mdpi.com/article/10.3390/ijms25158158/s1.
