# Supplementary material for: Telomere Length, Telomerase Activity, and Vaginal Microbiome in Patients with HPV-Related Precancerous Lesions
Source: Int J Mol Sci. 2024 Jul 26;25(15):8158. doi: 10.3390/ijms25158158 (PMC11311766; doi:10.3390/ijms25158158)
Supplement: Supplementary file 1 [file ijms-25-08158-s001.zip › ijms-3093255-supplementary.pdf]

Supplementary materials

Table S1. Biocenosis quality in disease stage prognosis

Disease stage	Biocenosis	Status of disease stage		Sensitivity (95% CI), % High to low	Specificity (95% CI), % High to low	PPV (95% CI), % High to low	NPV (95% CI), % High to low	Accuracy (95% CI), % High to low
		+	-					
**Carriers** (vs control)	Dysbiosis/*L.iners*	17	2	56.67 (37.43-74.54)	83.33 (51.59-97.91)	89.47 (69.78-96.90)	43.48 (32.22-55.45)	64.29 (48.03-78.45)
	Optimal	13	10					
**L SIL** (vs carriers)	Dysbiosis/*L.iners*	11	17	78.57 (49.20-95.34)	43.33 (25.46-62.57)	39.29 (29.92-49.51)	81.25 (59.46-92.76)	54.55 (38.85-69.61)
	Optimal	3	13					
**H SIL** (vs L SIL)	Dysbiosis/*L.iners*	39	11	84.78 (71.13-93.66)	21.43 (4.66-50.80)	78.00 (72.43-82.71)	30.00 (11.30-59.04)	70.00 (56.79-81.15)
	Optimal	7	3					
**Cancers** (vs H SIL)	Dysbiosis/*L.iners*	7	39	100.00 (59.04-100.00)	15.00 (6.34-28.87)	15.22 (13.70-16.87)	100.00 (59.04-100.00)	26.42 (15.26-40.33)
	Optimal	0	7					

PPV – positive predictive value, NPV – negative predictive value. Colour coding: highest to lowest value in each column.
